# Compound Heterozygous Variants in a Surviving Patient With Alkuraya-Kučinskas Syndrome: A New Case Report and a Review of the Literature

**DOI:** 10.3389/fped.2022.806752

**Published:** 2022-03-04

**Authors:** Ling Yue, Mei Jin, Xin Wang, Jing Wang, Ling Chen, Rong Jia, Zuozhen Yang, Fan Yang, Jingman Li, Cuiying Chen, Huacheng Zheng, Huafang Yang

**Affiliations:** ^1^Department of Neurological Rehabilitation, Children's Hospital of Hebei Province, Hebei, China; ^2^Electrophysiology Room, Children's Hospital of Hebei Province, Hebei, China; ^3^Department of Neurology, Children's Hospital of Hebei Province, Hebei, China; ^4^Cipher Gene LLC, Beijing, China

**Keywords:** KIAA1109, Alkuraya-Kučinskas syndrome, compound heterozygous variants, survival, autosomal recessive, club foot

## Abstract

**Background:**

Alkuraya–Kučinskas syndrome is an autosomal recessive disorder characterized by brain abnormalities associated with cerebral parenchymal underdevelopment, arthrogryposis, club foot, and global developmental delay. Most reported cases were cases of premature termination of pregnancies or neonatal deaths. To date, limited studies of nine surviving patients with global developmental delay and intellectual disability have been reported. In this study, we report another surviving patient.

**Methods:**

Whole-exome sequencing was utilized for the proband, and variants were filtered, annotated, and classified. Candidate variants were validated by Sanger sequencing of the proband and his family. The literature was reviewed; the prognosis among different regions and the variant type was analyzed.

**Results:**

A non-synonymous variant [NM_015312.3: exon29: c.4892C>G (p.Pro1631Arg)] was identified and validated in the patient's father. A frameshift duplication [NM_015312.3: exon62: c.10872dupA (p.Arg3625Lysfs^*^5)] that caused early translation termination was identified in his mother. The literature was reviewed, variants were classified into three regions of KIAA1109, and their survival status was summarized.

**Conclusion:**

We reported another survival proband with Alkuraya–Kučinskas syndrome driven by KIAA1109. Our case expands the genotypic spectrum of Alkuraya–Kučinskas syndrome and explored the relationship between the variant region and survival.

## Introduction

Alkuraya-Kučinskas syndrome (ALKKUCS) is a severe neurodevelopmental disorder characterized by global developmental delay, brain abnormalities, and arthrogryposis. Most probands die from the embryo suspension or soon after birth, and patients who survive always suffer from intellectual disabilities and epilepsy (1).

KIAA1109 was first cloned and sequenced using a size-fractionated adult brain cDNA (complementary DNA) library (2). Khuong et al. (3) demonstrated that tweek (the fly orthologue of KIAA1109) plays a crucial role in the growth of synapses at neuromuscular junctions (NMJs) by controlling both an Nwk (FCHSD2)-dependent pathway and phosphatidylinositol 4,5-bisphosphate [PI(4,5)P2] and Wsp-dependent pathways.

In 2015, another study identified a homozygous non-sense mutation in the KIAA1109 gene from a female infant in a consanguineous Saudi family (4). In 2018, Gueneau et al. (1) reported more homozygous or compound heterozygous mutations in 12 patients from nine unrelated families with ALKKUCS. Among the 13 patients reported by Gueneau et al. (1), only three patients from two families were alive at 7, 11, and 13 years of age. All patients had a global developmental delay from infancy and variable levels of intellectual disability. Besides, another two survival patients with severe phenotype were reported in 2019 (5). In 2020, Kumar et al. described four other surviving patients from two related families, similar to the cases reported by Gueneau et al. all of whom also suffered from global developmental delay and mild-to-severe intellectual disability (6).

To date, limited studies of surviving patients with global developmental delay and intellectual disability have been reported. In this study, we report another surviving patient carrying new variants in KIAA1109 and explore the potential relationship between the variant region and survival status.

## Materials and Methods

### Proband

Informed consent was obtained from the patient's family members. This study was approved by the Institutional Review Board of the Children's Hospital of Hebei Province. The data on his clinical features, electroencephalogram (EEG), brain magnetic resonance imaging (MRI), malformations, and other examination results were collected.

### WES and Sanger Sequencing

Genomic DNA was extracted from whole-blood samples. The IDT (Integrated DNA Technologies, USA) XGen Exome Research Panel was used to capture libraries, and the library was sequenced on the Novaseq 6,000 Sequencing platform. Finally, the paired end clean reads were mapped to the human reference genome (GRCh38/hg38). Variations were annotated using ANNOVAR software (7), and SNPs (Single Nucleotide Polymorphism) with a minor allele frequency ≤0.005 in the SNP database were obtained for further analysis. The pathogenic evaluation was performed according to the American College of Medical Genetics and Genomics (ACMG) guidelines (8). The Sanger sequencing of candidate variants was performed in the proband and his parents to validate the variation identified by whole-exome sequencing.

## Results

### Case Presentation

The infant was a boy of the second pregnancy of a healthy 28-year-old mother. He was born at full term (39 weeks gestational age) natural delivery, weighing 3.7 kg. After birth, routine physical examination revealed a normal result. By about 3 months of age, failure to thrive was observed including failing to hold his head and turn over. At 4 months of age, he developed intermittent limb clonus and was admitted to a hospital.

**Figure 1:**
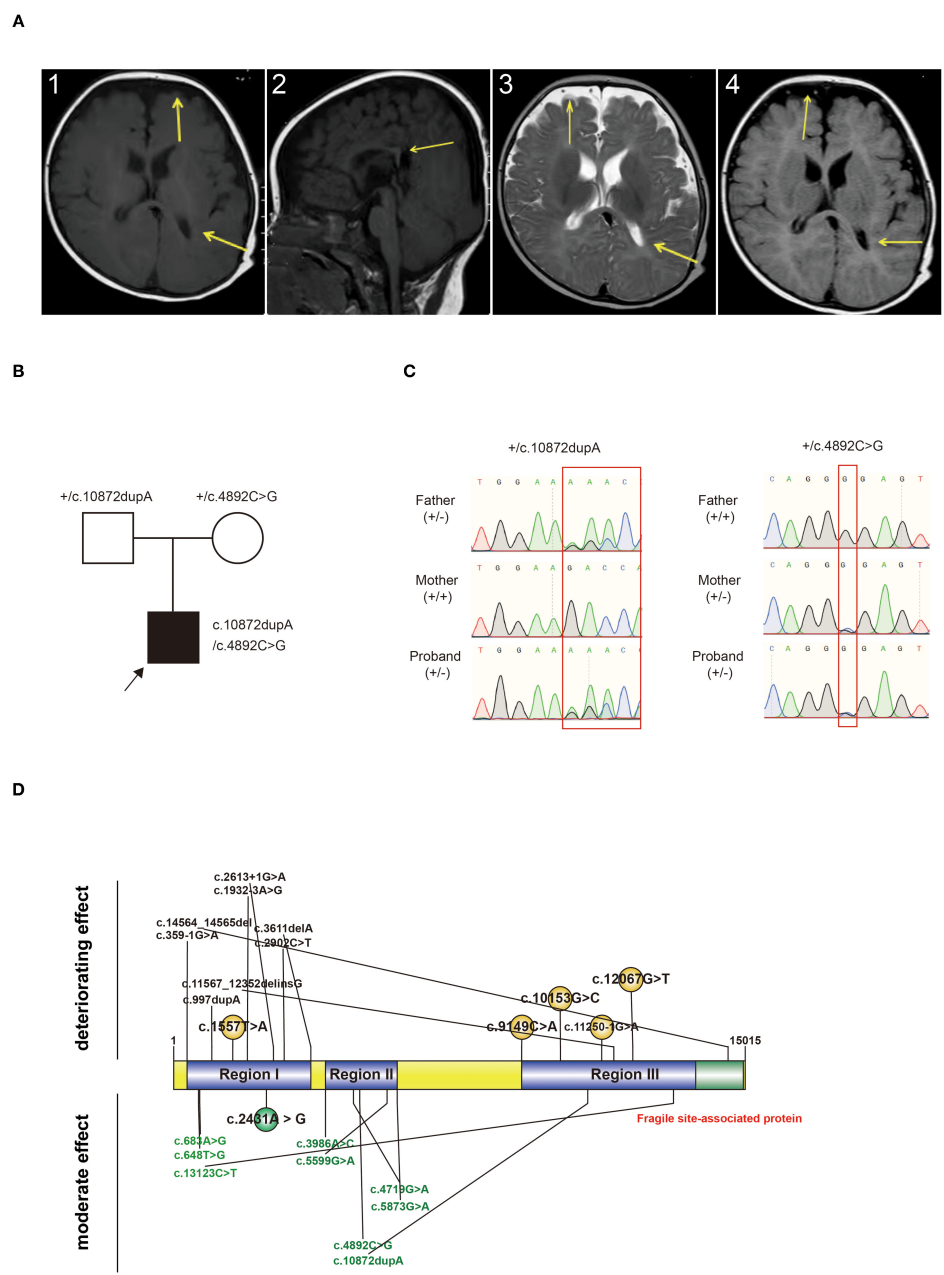
**(A)** Characteristic changes in the MRI findings of the proband. MRI images revealed a widening of bilateral frontotemporal space, an irregular shape of the left ventricle, and a short corpus callosum. A.1 and A.2: T1W; A.3: T2W; A.4: FLAIR. Arrow: Change of sites. MRI: magnetic resonance imaging. **(B)** Pedigree chart and genotype information of the proband. Black arrow: proband. +: wildtype. **(C)** Genotype validated by Sanger sequencing. Compound heterozygous variants were inherited from both the father and mother. +: wildtype; -: variant type. **(D)** Variant-reported summary. Variants from one patient were labeled together. Variants written in black are those from dead probands. Variants written in green are those from probands who survived. Variants written in red are those discovered in our patient.

Routine blood, homocysteine, genetic metabolism screening, and physical examination demonstrated normal results. Also, no abnormality was found in otolaryngology and ophthalmic examination. Cranial MRI showed that bilateral frontotemporal space was widened, the left ventricle was irregular, and the corpus callosum was short (Figure 1A). Video EEG revealed a normal result.

Convulsions could be observed in genetic metabolic disease, electrolyte disorder, and genetic disease. We excluded metabolic disease by negative results of genetic metabolic screening, excluded electrolyte disorder by the absence of typical clinical features including diarrhea, vomiting, poor eating, and other manifestations. Finally, we uncovered the candidate mutations in the KIAA1109 gene.

We treated the patient with oxiracetam (1 g/day for 10 days), cerebroside (2 ml/day for 10 days), and carnosine (0.1 g/day for 7 days); observed that the limb clonus disappeared; and then discharged him.

We followed up the patient when he was 2 years and 7 months old; he still showed global development delay, unstable walking, abnormal posture, foot valgus, poor balance ability, poor speech, and cognitive ability.

### Exome Sequencing and Analysis

WES detected compound heterozygous variants in KIAA1109 (Figure 1B). The first variant [c.4892C>G (p.Pro1631Arg)] was a non-synonymous variant, causing amino acids from proline to arginine at 1,631st of the protein. The second variant was a frameshift duplication insertion at 3,625th of KIAA1109, causing amino acid translation from arginine to lysine, and terminating at 5th after the variant site. Both variants were absent in published databases, such as gnomeAD and Exome Aggregation Consortium (ExAC). c.4892C>G was predicted to cause damage to proteins (SIFT: Damaging, Polyphen2 _HDIV: Damaging, Mutation Taster: Disease-causing). Both variants were classified as VUS or LP according to the ACMG guidelines (Table 1). Both variants were validated from the father and mother, respectively, by Sanger sequencing (Figure 1C).

**Table 1:** Analysis of variants detected in KIAA1109 (NM_015312.3).

**No**.	**Variants**	**AA change**	**GnomeAD MAF**	**Inherit**	**Classification**	**Evidence**
1	c.4892C>G	p.Pro1631Arg	Absent	Mother	VUS	PM2_Supporting+PM3 + PP3
2	c.10872dupA	p.Arg3625Lysfs*5	Absent	Father	LP	PVS1 + PM2_Supporting

*MAF, minor allele frequency; VUS, variant of uncertain significance; LP, likely pathogenic*.

We reviewed all reported variants and survival information and defined three regions (Region I: 358–3,611 bp; Region II: 3,986 bp−5,873 bp; Region III: 9,149–14,564 bp) among full-length KIAA1109 (Figure 1D). We found that homozygous or compound heterozygous variants in regions I and III had a deteriorating effect on the patients, whereas compound heterozygous variants in region II showed a moderate effect on survival (Figure 1D). Interestingly, the variants for our survival case were compound heterozygous variants from regions II and III.

In order to explore a potential relationship between variant type (missense, non-sense, splice site, frameshift) and survival status, we combined non-sense, splicing, and frameshift as structure variants since they resulted in early premature termination and deletion exon or insertion intron. As an autosomal recessive disorder, bi-allelic variants were classified into three groups: missense+missense; missense+structure; and structure+structure. We reviewed all variants and the survival status of reported cases and found that 60% (9/15) (missense + missense) patients survived, and 40% (6/15) (missense + missense) patients died; 33.3% (1/3) (missense + structure) patients survived, and 66.7% (2/3) (missense + structure) patients died; and 100% (4/4) (structure+structure) patients died (Table 2). However, there was no significant relationship between the variant and survival status (*p* > 0.05).

**Table 2:** Phenotype related to variant type.

	**Moderate effect**	**Deteriorating effect**
Missense + missense	60% (9/15)	40% (6/15)
Missense + structure	33.3% (1/3)	66.7% (2/3)
Structure + structure	0% (0/4)	100% (4/4)

Among 10 survival patients, 60 (6/10) presented severe global developmental delay, 20 (2/10) moderate delay, and 20% (2/10) mild delay. A certain proportion of organs exhibited dysfunction including 60 (6/10) in head and faces, 20 (2/10) in eyes, 20 (2/10) in mouth, 30 (3/10) in joints, 60 (6/10) in limbs, 40 (4/10) in gastrointestinal tract, 10 (1/10) in urogenital tract, 30 (3/10) in heart, 20 (2/10) in muscle, and 80% (8/10) in behavior. Development delay and abnormality in behavior, head, and face were widely observed in survival patients (Supplementary Material Table). Interestingly, our patient showed relative mild phenotype by only presenting development delay, limbs clonus, unstable walking, and foot valgus. Malfunctions in head and faces, eyes, mouth, gastrointestinal, urogenital, heart, and muscle were not observed.

## Discussion

Kiaa1109, located on the long arm of chromosome 4 (4q27), contains 84 exons and 15,592 base pairs in length (https://www.ncbi.nlm.nih.gov/nuccore/NM_015312.3). It is widely expressed in many different tissues in humans as viewed by BioGPS (9) and is predominantly expressed in the parathyroid, muscles, ears, eyes, mammary glands, lymph nodes, thymus, and 27 other tissues. KIAA1109 is also involved in various tumors such as bladder carcinoma, chondrosarcoma, glioma, leukemia, lymphoma, non-neoplasia, and retinoblastoma tissues.

KIAA1109 is conserved in many species. Orthologs have been found in many mammals and other vertebrates, and homologs have been identified in animals (such as insects). No human paralogs for KIAA1109 have been identified (10). The mRNA sequence identity in mammals ranges from 81.9 (platypus) to 99.5% (chimpanzees). In addition, the protein identity ranged from 93.2 in opossum to 99.8% in chimpanzees, and protein similarity was no less than 97% in all mammals. In addition, birds continue to show fairly high conservation with protein identities of ~90 and protein similarities of 96%.

The NCBI conserved domain search identified two potential domains in KIAA1109 (11). The first is the fragile site around the C-terminus, which is associated with celiac disease susceptibility according to genome-wide association studies and may also be associated with polycystic kidney disease. The second conserved region is an uncharacterized conserved protein (DUF2246), whose function is unknown and conserved in various species, from humans to worms. It also contains one transmembrane domain from amino acids 26–46 (https://www.ncbi.nlm.nih.gov/protein/150378498). No signal peptides, mitochondrial targeting sequences, or chloroplast peptides were predicted; therefore, there was no localization to the secretory pathway, mitochondria, or chloroplast.

The correlation between variants and prognosis is not well-established as a severe neurodevelopmental and fetal disorder. In this study, we classified all reported patients and their variants and found that homozygous or compound heterozygous variants in region I or region III lead to deteriorating effects for the probands, whereas compound heterozygous variants in region II showed moderate effects on survival. The only exception is a proband carrying a homozygous variant of c.2431A>G in region I. Our cases carry one variant from regions II and III, conforming compound heterozygous variants. In order to understand the potential underlying mechanism between the region and survival status, we searched the domain information in SWISS-MODEL (https://swissmodel.expasy.org/repository/uniprot/Q2LD37), InterPro (https://www.ebi.ac.uk/interpro/protein/UniProt/Q2LD37/), and UniProt (https://www.uniprot.org/uniprot/Q2LD37), found a fragile site-associated region at C-terminal of KIAA1109 that shared 468 amino acids with region III and a transmembrane helical from the 26th to 46th amino acid at N-terminal that does not share any overlap with our variants. Due to the limited domain information, it was hard to draw any conclusion from the relationship between a variant and functional domain.

Also, due to the limited number of reported cases with variants and prognoses, the region definition may be inaccurate. We believe it could be modified more accurate with additional cases in the future.

## Conclusion

In this study, we report the eighth surviving patient with ALKKUCS who carries new variants in KIAA1109 and explore the potential relationship between variant regions and survival status. Our report extended the understanding of the ALKKUCS genotype and survival cases and provided more evidence of a better prognosis for ALKKUCS syndrome.

## Data Availability Statement

The original contributions presented in the study are included in the article/Supplementary Materials, further inquiries can be directed to the corresponding author/s.

## Ethics Statement

The studies involving human participants were reviewed and approved by Institutional Review Board of the Children's Hospital of Hebei Province. Written informed consent to participate in this study was provided by the participants' legal guardian/next of kin.

## Author Contributions

LY: conceptualization, methodology, and data mining. XW, JW, JL, CC, HZ, and HY: writing—original draft preparation. LC and RJ: clinical data collection. ZY and FY: software, data mining, and investigation. MJ: supervision, writing—reviewing and editing. All authors contributed to the article and approved the submitted version.

## Funding

This work was supported by the Hebei Medical Science Research Project (No. 20190785).

## Conflict of Interest

ZY and FY were employed by CipherGene LLC. The remaining authors declare that the research was conducted in the absence of any commercial or financial relationships that could be construed as a potential conflict of interest.

## Publisher's Note

All claims expressed in this article are solely those of the authors and do not necessarily represent those of their affiliated organizations, or those of the publisher, the editors and the reviewers. Any product that may be evaluated in this article, or claim that may be made by its manufacturer, is not guaranteed or endorsed by the publisher.
